# Lack of Prion Accumulation in Lymphoid Tissues of *PRNP* ARQ/ARR Sheep Intracranially Inoculated with the Agent of Scrapie

**DOI:** 10.1371/journal.pone.0108029

**Published:** 2014-09-18

**Authors:** Justin J. Greenlee, Robert A. Kunkle, Jürgen A. Richt, Eric M. Nicholson, Amir N. Hamir

**Affiliations:** Virus and Prion Research Unit, National Animal Disease Center, Agricultural Research Service, United States Department of Agriculture, Ames, Iowa, United States of America; Creighton University, United States of America

## Abstract

Sheep scrapie is a transmissible spongiform encephalopathy that can be transmitted horizontally. The prion protein gene (*PRNP*) profoundly influences the susceptibility of sheep to the scrapie agent and the tissue levels and distribution of PrP^Sc^ in affected sheep. The purpose of this study was to compare the survival time and PrP^Sc^ tissue distribution in sheep with highly resistant and highly susceptible *PRNP* genotypes after intracranial inoculation of the agent of scrapie. Five sheep each of genotype VRQ/VRQ, VRQ/ARR or ARQ/ARR were inoculated. Sheep were euthanized when clinical signs of scrapie became severe. Clinical signs, microscopic lesions, and western blot profiles were uniform across genotypes and consistent with manifestations of classical scrapie. Mean survival time differences were associated with the 171 polymorphic site with VRQ/VRQ sheep surviving 18 months, whereas VRQ/ARR and ARQ/ARR sheep survived 60 and 56 months, respectively. Labeling of PrP^Sc^ by immunohistochemistry revealed similar accumulations in central nervous system tissues regardless of host genotype. Immunoreactivity for PrP^Sc^ in lymphoid tissue was consistently abundant in VRQ/VRQ, present but confined to tonsil or retropharyngeal lymph node in 4/5 VRQ/ARR, and totally absent in ARQ/ARR sheep. The results of this study demonstrate the susceptibility of sheep with the ARQ/ARR genotype to scrapie by the intracranial inoculation route with PrP^Sc^ accumulation in CNS tissues, but prolonged incubation times and lack of PrP^Sc^ in lymphoid tissue.

## Introduction

Scrapie is a horizontally transmitted, uniformly fatal neurodegenerative disease of sheep and goats. Definitive diagnosis is typically defined by postmortem pathology and detection of the disease-specific prion protein, called PrP^Sc^, in affected tissues. [1] Most scrapie infections are presumably acquired by the oral route. Exposure to the scrapie agent initiates an autocatalytic, templated conversion of the highly conserved, host-encoded, membrane-anchored glycoprotein PrP^C^ to the abnormally folded PrP^Sc^. The clinical and pathological hallmarks of scrapie develop as PrP^Sc^ gradually accumulates in the central nervous system (CNS) months or years after initial exposure.

At the clinical stage of scrapie, PrP^Sc^ is widely distributed throughout the CNS and is associated with microscopic evidence of neuronal vacuoles, neuronal loss, astrocytosis, and generalized vacuolation of the neuropil. Lymphoid tissues also can accumulate PrP^Sc^ in scrapie-affected sheep, and immunohistochemical (IHC) detection of PrP^Sc^ in palatine tonsil, [2] third eyelid, [3] or gut-associated lymphatic tissue of the rectal mucosa [4] of sheep has been used for the diagnosis of scrapie at either the preclinical or clinical stage of disease.

The susceptibility of sheep to scrapie is greatly influenced by host prion protein gene (PRNP) alleles. Amino-acid polymorphisms corresponding to the codons 136, 154 and 171 are major determinants of relative susceptibility or resistance. The five common allelic variations on codons 136, 151, and 171 result from amino-acid substitutions involving alanine (A), valine (V), arginine (R), histidine (H) and glutamine (Q), are A_136_R_154_R_171_, A_136_R_154_H_171_, A_136_H_154_Q_171_, A_136_R_154_Q_171_ and V_136_R_154_Q_171_. [5] The V_136_ and Q_171_ haplotypes are linked to scrapie susceptibility, especially in the homozygous state. [6]–[8] Although the predictive value of *PRNP* genotype in determining the outcome of exposure to the scrapie agent is not absolute, the VRQ/VRQ genotype present in certain sheep breeds such as Cheviot is strongly associated with susceptibility and the ARR/ARR genotype is strongly associated with resistance. [6], [9], [10] Therefore, PRNP polymorphisms found within a regional or national flock can be used for selective breeding recommendations to reduce the incidence of scrapie [11], [12].

Classical scrapie can be transmitted to genetically susceptible sheep, but many attempts to transmit the disease by oral inoculation of ARQ/ARR sheep have not been successful. [13]–[15] Intracranial (IC) inoculation of animals with TSE infected material consistently produces prion disease in compatible recipients and can result in PrP^Sc^ accumulation in hosts thought to be resistant by natural routes. For example, intracranial inoculation, but not oral inoculation results in transmission of scrapie to cattle [16], [17] and transmission of bovine spongiform encephalopathy (BSE) to swine, albeit without classical clinical signs of TSE [18].

The purpose of this study was to compare the survival time and PrP^Sc^ tissue distribution in sheep with highly resistant and highly susceptible *PRNP* genotypes after intracranial inoculation of the agent of scrapie.

## Materials and Methods

### Genotyping

Prion protein amino acid sequences were determined as described previously [19]. Primers specific for the functional gene in sheep (forward primer Sheep-F2: 5′- GGA GTG ACG TGG GCC TCT GC -3′ and reverse primer Sheep-R4: 5′- CTC CCT CCC CCA ACC TGG CA -3) amplified a 775-bp region beginning at codon 19 in the PRP coding region in sheep. All PCR reactions were as follows: 95°C for 5 min, followed by 30 cycles of denaturation (95°C, 20 sec), annealing (60°C, 20 sec) and extension (72°C, 60 sec) followed by an extension cycle (72°C, 7 min). Following filtration, PCR products were sequenced using primers Sheep-F2, Sheep-F3 (5′- ATG GAG GTG GCT GGG GCC AA -3), Sheep-R3 (5′- TCC CCC TTG GTG GTG GTG GT -3) and Sheep-R4. Resulting sequences were analyzed using Geneious version 6, created by Biomatters. Available from http://www.geneious.com/.

### Experimental Animals

This experiment was carried out in accordance with the Guide for the Care and Use of Laboratory Animals (Institute of Laboratory Animal Resources, National Academy of Sciences, Washington, DC) and the Guide for the Care and Use of Agricultural Animals in Research and Teaching (Federation of Animal Science Societies, Champaign, IL). The protocol was approved by the Institutional Animal Care and Use Committee at the National Animal Disease Center (protocol numbers: 3219 and 3281). Fifteen four-month-old lambs (Cheviot and Suffolk) of different *PRNP* genotypes (at codons 136, 154, 171) from a scrapie-free flock were obtained for this study. The lambs were divided by genotypes into three groups (A, B and C) and inoculated by the intracranial (IC) route while under injectable (xylazine) anesthesia. Group A (n = 5) was composed of VRQ/VRQ (Cheviot) sheep; group B (n = 5) was VRQ/ARR (Cheviot) and group C (n = 5) was ARQ/ARR (Suffolk) (Table 1). All animals were IC inoculated with inoculum (no. 13-7) that has been previously demonstrated to be infectious by IC and oral routes to Suffolk which are ARQ/ARQ or VRQ/ARQ at codons 136, 154, and 171 of *PRNP* gene, respectively, [20] and utilized in a number of additional transmission studies [12], [14], [21]–[26].

**Table 1:** 

Genotype	survival time (MPI)	spongiform encephalopathy	Immunohistochemistry (IHC)		
		CNS	CNS	Lymph	Lymph
Group (A) Cheviot				A* B†	(all)
VRQ/VRQ (n = 5)		(5/5)	(5/5)	(5/5) (5/5)	(28/29)
T22	17	+	+	+ +	(5/5)
53	18	+	+	+ +	(6/6)
T23	18	+	+	+ +	(6/6)
57	18	+	+	+ +	(6/6)
6	17	+	+	+ +	(5/6)
group mean	18				
Group (B) Cheviot					
VRQ/ARR (n = 5)		(5/5)	(5/5)	(4/5) (0/5)	(5/30)
68	65	+	+	+ −	(1/6)
56	60	+	+	+ −	(1/6)
64	57	+	+	− −	(0/6)
67	59	+	+	+ −	(1/6)
62	59	+	+	+ −	(2/6)
group mean	60				
Group (C) Suffolk					
ARQ/ARR (n = 5)		(4/5)	(5/5)	(0/5) (0/5)	(0/30)
053	46	−	+	− −	(0/6)
054	60	+	+	− −	(0/6)
057	46	+	+	− −	(0/6)
050	65	+	+	− −	(0/6)
055	65	+	+	− −	(0/6)
group mean	56				

MPI = months post-inoculation; CNS = central nervous system; Lymph = lymphoid tissues; IHC = immunohistochemistry;

*Lymphoid A: retropharyngeal lymph node and palatine tonsil.

† Lymphoid B: spleen, pharyngeal tonsil, mesenteric lymph node, and ileal Peyer’s patches.

Briefly, the inoculum no. 13-7 was prepared from a pool of brains from sheep confirmed immunoreactive for PrP^Sc^ by the United States Department of Agriculture.

Animal and Plant Health Inspection Services National Veterinary Services Laboratory. [20] The sheep were from various regions of the U.S., but were all ARQ/ARQ genotype. The homogenate (10% wt/vol) of infected brains was prepared in phosphate buffered saline (PBS) containing gentamicin using a mechanical grinder. All animals were inoculated IC with 1 ml of the inoculum as described previously [27].

Inoculated animals were housed in a biosafety level-2 containment facility for 2 weeks post-inoculation and later were moved to outside pens at NADC. The sheep were fed pelleted growth and maintenance rations that contained no ruminant protein and clean water was available *ad lib*.

### Clinical and post-mortem examination

The sheep were observed twice daily throughout the experiment for development of clinical signs. All sheep were euthanized by intravenous pentobarbital overdose when they developed unequivocal neurologic signs consistent with scrapie: ataxia, tremors, incoordination, or reluctance or inability to rise. Carcasses were examined at necropsy and two sets of tissue samples were collected. One set of tissues included representative sections of lymphoid tissues (spleen, pharyngeal and palatine tonsil, retropharyngeal and mesenteric lymph node, and ileal Peyer’s patches), liver, kidney, skin, striated muscles (heart, tongue, diaphragm, masseter), thyroid gland, nasal turbinates, lung, small intestine (jejunum and ileum), adrenal gland, pituitary gland, trigeminal ganglion, brain (hemisections at the levels of cerebral cortex, cerebellum, superior colliculus and brainstem including obex) and eye (retina). These tissues were fixed in 10% buffered formalin, embedded in paraffin wax, sectioned at 5 µm, and stained with hematoxylin and eosin (HE) for light microscopy. The second set of tissues was frozen.

### Western blot

One half of brain (cut longitudinally) was frozen for western blot (WB) as described previously. [28] Portions of brainstem from each sheep were sampled for WB examination. Briefly, monoclonal P4 (R-Biopharm, Inc., Marshall, MI) as primary antibody and biotinylated anti-mouse (dilution 1∶10,000; Biotinylated anti-mouse IgG, Amersham Biosciences, USA) as secondary antibody, followed by streptavidin horseradish-peroxidase conjugate (dilution 1∶10.000; Streptavidin horseradish-peroxidase conjugate, Amersham Biosciences, USA) were utilized. The immunoblot was developed with a chemiluminescence solution (ECL Plus, Amersham Biosciences, USA) before being scanned by an imaging system.

### Immunohistochemistry

All paraffin embedded tissues were stained by an automated immunohistochemical (IHC) method for detection of PrP^Sc^ as described previously. [20] Briefly, following deparaffinization and rehydration, tissue sections were autoclaved for 30 minutes in an antigen retrieval solution (DAKO Target Retrieval Solution, DAKO Corp., Carpinteria, CA) and stained with an indirect avidin biotin system (Basic Alkaline Phosphatase Red Detection Kit, Ventana Medical Systems, Inc., Tucson, AZ) designed for an automated immunostainer (NexES IHC module, Ventana Medical Systems, Inc., Tucson, AZ). A cocktail of 2 primary monoclonal antibodies was used: F89/160.1.5 and F99/97.6.1, each at a concentration of 5 µg/ml. Incubation was carried out at 37°C for 32 min. The secondary antibody was biotinylated anti-mouse (Biotinylated anti-mouse IgG (made in horse), Vector Laboratories, Burlingame, CA) diluted 1∶200 and incubated for 8 min at 37°C [20].

## Results

As noted above, sheep were assigned to groups based on polymorphism positions 136 (V/A) and 171 (Q/R). Genotyping results showed all sheep to be homozygous at positions 112M, 127G, 137M, 138S, 141L, 151R, 154R, 157M, 176N, 180H, 189Q, 195T, 196T, 211R, 220Q, 223R.

All inoculated sheep (15/15) developed clinical scrapie and were euthanized between 17 and 65 MPI (Table 1). Cheviots with genotype VRQ/VRQ (5/5) developed the disease early and were euthanized between 17 and 18 MPI (mean = 18 MPI), whereas Cheviots with genotype VRQ/ARR (5/5) were euthanized later between 56 and 65 MPI (mean = 60 MPI). Suffolks with genotype ARQ/ARR (5/5) had approximately similar survival time with a mean of 56 MPI, being euthanized between 46 and 65 MPI (Table 1).

Clinical signs of affected sheep consisted of a progressive decrease in appetite with associated weight loss. Later, the affected sheep developed fine head tremors, listlessness, progressive problems with locomotion, and terminal sternal recumbency. None of the affected sheep exhibited obvious pruritus or loss of wool from their fleece. Apart from fair to poor nutritional condition of the carcasses, no significant gross lesions were seen in any of the sheep examined.

**Figure 1:**
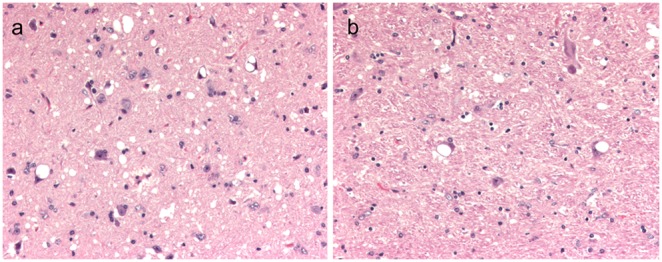
Vacuolar change in ARQ/ARR sheep. (**A**) Section of midbrain at the level of the superior colliculus from sheep 050 with multifocal neuronal vacuolation and extensive spongiform change in neuropil. (**B**) Section of brainstem at the level of the obex from sheep 055 with large intraneuronal vacuoles. Hematoxylin and Eosin Bar = 50 µm.

**Figure 2:**
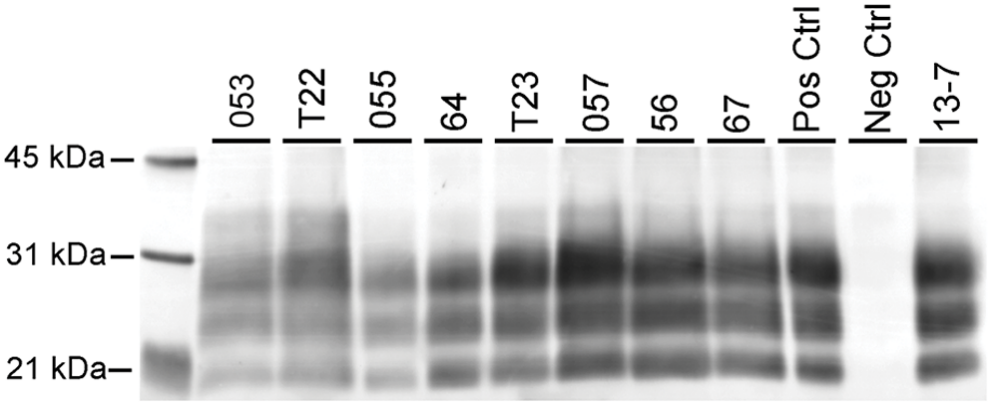
Western blot analysis demonstrating similar PrP^Sc^ profile in brainstem regardless of genotype when developed with mAb P4. Note uniform 3-band molecular profile identical to inoculum (13-7) profile; individuals of VRQ/VRQ (sheep T22, T23), VRQ/ARR (sheep 64, 56, 67), ARQ/ARR (sheep 053, 055, 057) prion genotypes represented (see Table 1). Samples were loaded at varying concentrations between 0.06–0.24 mg equivalents of brain tissue per lane. Inoculum (No. 13-7) was loaded at 0.06 mg and the positive control was loaded at 0.03 mg. Molecular weight markers in kDa are indicated on the left side of the blot.

**Figure 3:**
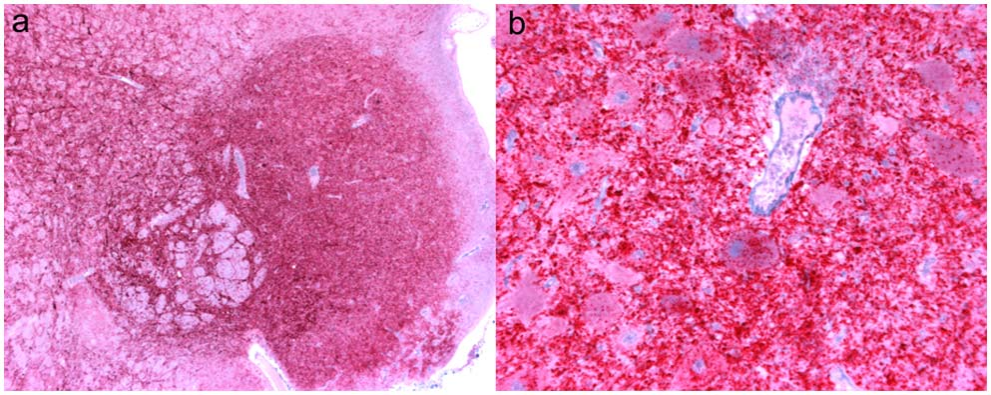
Immunoreactivity (red) is abundant in ARQ/ARR sheep in brainstem at the level of the obex. (**A**) Low magnification view of brainstem at the level of the obex from sheep 050 demonstrates extensive PrP^Sc^ immunoreactivity. Bar = 500 µm. (**B**) High magnification of brainstem from sheep 050 with abundant PrP^Sc^ immunoreactivity around and within neuronal perikarya and throughout the neuropil. PrP^Sc^ immunohistochemistry with monoclonal antibodies F89/160.1.5 and F99/97.6.1 and hematoxylin counterstain. Bar = 50 µm.

**Figure 4:**
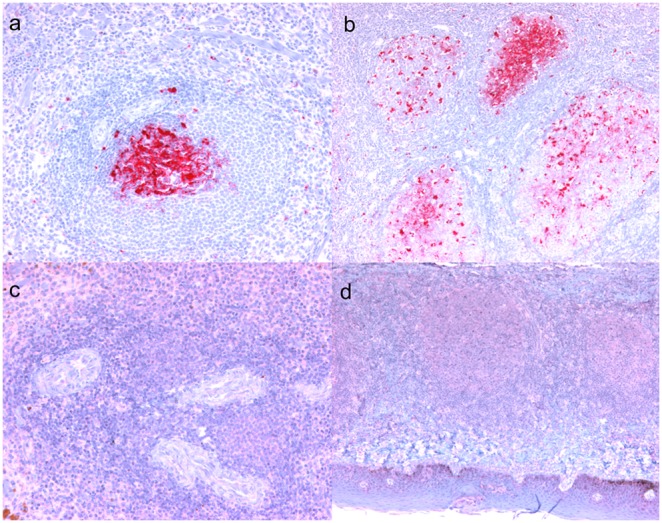
Presence of PrP^Sc^ immunoreactivity in lymphoid tissues after intracranial inoculation is associated with genotype. (**A**) Spleen from a VRQ/VRQ (sheep 057) with confluent PrP^Sc^ immunoreactivity in a lymphoid follicle germinal center adjacent to a periarteriolar lymphoid sheath. (**B**) Palatine tonsil from a VRQ/VRQ sheep (sheep 057) with PrP^Sc^ immunoreactivity of germinal centers of lymphoid follicles. (**C**) Spleen from an ARQ/ARR sheep (sheep 050) devoid of immunoreactivity in the periarteriolar lymphoid sheaths. (**D**) Palatine tonsil from an ARQ/ARQ sheep (sheep 050) lacking immunoreactivity in submucosal lymphoid follicles. PrP^Sc^ immunohistochemistry with monoclonal antibodies F89/160.1.5 and F99/97.6.1 and hematoxylin counterstain. Bar = 50 µm (a,c); bar = 140 µm (b,d).

**Figure 5:**
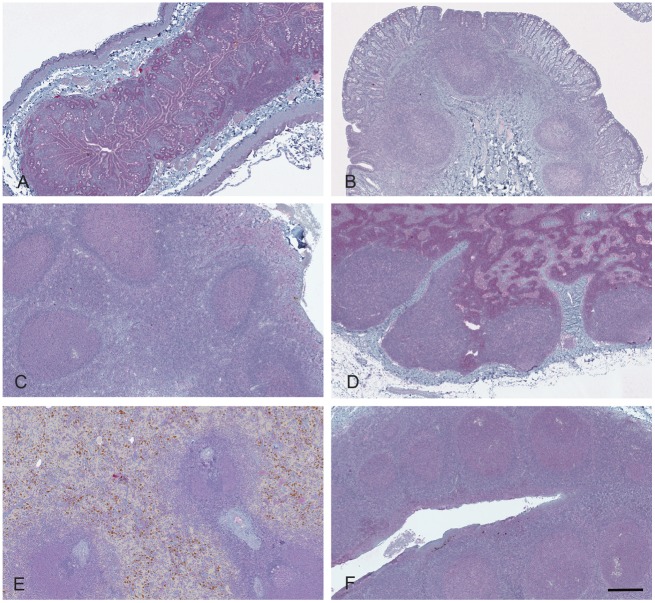
Lack of immunoreactivity in lymphoid tissues of an ARQ/ARR sheep (sheep 050) after intracranial inoculation. The lymphoid follicles of (**A**) ileal Peyer’s patches, (**B**) gut associated lymphatic tissues of the recto-anal junction, (**C**) pharyngeal tonsil, (**D**) retropharyngeal lymph node, (**E**) spleen, and (**F**) palatine tonsil are devoid of PrP^Sc^ immunoreactivity after immunohistochemistry with monoclonal antibodies F89/160.1.5 and F99/97.6.1 and hematoxylin counterstain. Brown pigment in spleen (**E**) is hemosiderin-laden macrophages in red pulp. Bar = 180 µm.

The presence of microscopic lesions (spongiform encephalopathy) and the results of PrP^Sc^ by IHC and WB assays are given in Table 1 and shown in Figs. 1–5. With the exception of a single ARQ/ARQ sheep (053) necropsied at 46 months PI that lacked spongiform change, severe spongiform lesions were consistently present in various regions of the brain with greatest intensity in the brainstem (obex) and colliculus and lesser intensity in cerebellum and cerebrum. The lesions consisted of vacuolar change in the neuropil and distinct vacuoles within neuronal perikarya (Fig. 1). They were most severe in the parasympathetic nucleus of the vagus nerve. In the affected areas there was a mild increase in the number of glial cells.

Western blot profiles (Fig. 2) were uniform across genotypes and demonstrated the characteristic 3-band pattern representing the di-, mono-, and non-glycosylated (approx. 21 kDa) forms of PrP^Sc^ present in classical scrapie.

Distribution of PrP^Sc^ immunoreactivity was similar in the central nervous system tissues regardless of host genotype (Table 1) and similar to previous studies. [20] The intensity of immunoreactivity appeared greatest in brainstem and colliculus and was present throughout the neuropil and within neuronal perikarya (Fig. 3). Sections of cerebellum and cerebrum examined also had PrP^Sc^ immunoreactivity except in a single ARQ/ARQ sheep (053) necropsied at 46 months PI. In this single animal, immunoreactivity was minimal and limited to the brainstem and colliculus. Retinal tissue was consistently immunoreactive for PrP^Sc^ in the plexiform layers with the exception of 1/5 VRQ/ARR and 2/5 ARQ/ARR sheep that had no detectable retinal PrP^Sc^.

Sheep genotype was associated with extent of immunoreactivity detected in lymphoid tissues. PrP^Sc^ immunoreactivity was consistently present in the lymphoid tissues of VRQ/VRQ sheep and was present in 4/5 VRQ/ARR sheep. However, all lymphoid tissues examined from ARQ/ARR sheep were completely devoid of PrP^Sc^ immunoreactivity (Table 1, Figs 4 and 5). Immunoreactivity for PrP^Sc^, where present, was localized predominately to germinal centers of lymphoid follicles. PrP^Sc^ was demonstrated in sections of palatine and nasopharyngeal tonsil, retropharyngeal and mesenteric lymph node, and spleen in all VRQ/VRQ sheep and the lymphoid tissue of Peyer’s patches of 3/4 VRQ/VRQ sheep with tissues available for examination. In VRQ/ARR sheep, PrP^Sc^ immunoreactivity was present only in palatine tonsil and/or retropharyngeal lymph node of 4/5 sheep (Table 1, Figs 4 and 5).

## Discussion

The purpose of this study was to compare the survival time and PrP^Sc^ tissue distribution in sheep with highly resistant and highly susceptible *PRNP* genotypes after intracranial inoculation of the agent of classical scrapie. Our results demonstrate that ARQ/ARR sheep are susceptible to scrapie following IC inoculation, but with extended survival times (relative to more susceptible genotypes) and absence of immunohistochemical evidence of PrP^Sc^ distribution within lymphoid tissues.

Clinical scrapie occurred in all genotypes inoculated in this study by the IC route: VRQ/VRQ, VRQ/ARR, and ARQ/ARR. Genetically resistant ARQ/ARR sheep had a mean incubation period of 56 months. This was markedly longer than the mean incubation of 18 months for genetically highly susceptible VRQ/VRQ sheep. Sheep of the ARQ/ARR genotype had a very similar mean incubation period to VRQ/ARR sheep, which was 60 months. Genotype of donor inoculum can play a role in scrapie incubation periods. [29] Prolonged incubation periods in VRQ/ARR sheep in the present study can be explained by the use of an inoculum pool composed of material from ARQ/ARQ sheep. This would be consistent with our previous studies using this inoculum where IC inoculated ARQ/ARQ sheep had an average incubation of 18 months, [20] which was the same as the VRQ/VRQ sheep reported in the present study. In contrast, experiments conducted with isolates of the scrapie agent from VRQ/VRQ sheep can have dramatically shorter incubation times when inoculated into VRQ/VRQ sheep: as short as 4.3 months PI after IC inoculation. [30] In QR171 sheep with scrapie, over 90% of the PrP^Sc^ is composed of the Q171 allelotype as the ARR allelotype has low conversion efficiency for PrP^Sc^. [31] Thus, the similarity in incubation time between ARQ/ARR and VRQ/ARR in the present experiment was not entirely unexpected.

Despite differences in incubation period, all genotypes of sheep had similar PrP^Sc^ immunoreactivity throughout the CNS. PrP^Sc^ was demonstrated by IHC in the CNS tissues in all IC inoculated sheep, but not in any of the lymphoid tissues collected from the ARQ/ARR sheep. In contrast, PrP^Sc^ was detected in nearly all lymphoid tissues collected from VRQ/VRQ sheep. Interestingly, VRQ/ARR sheep had detectable PrP^Sc^ only in palatine tonsil and/or retropharyngeal lymph node, but not peripheral lymphoid tissues such as Peyer’s patches, mesenteric lymph node, or spleen. It is not surprising that IHC of the lymphoid tissues of VRQ/VRQ sheep in the present study revealed abundant PrP^Sc^. In previously reported studies, oral inoculation of susceptible lambs results in detectable PrP^Sc^ in intestinal Peyer’s patch within 3 weeks and widespread in the lymphoid tissues from 5 weeks PI onward. [32], [33] The findings of the present study correspond to genetic susceptibility for scrapie: [12] immunoreactivity was abundant in lymphoid tissues of VRQ/VRQ sheep, absent in ARQ/ARR sheep, and intermediate in VRQ/ARR sheep (Table 1). The findings in VRQ/ARR sheep were similar to our previous results in ARQ/ARQ IC inoculated sheep where only a subset lymphoid tissues had detectable PrP^Sc^. [20] Preclinical and postmortem examination of LRS tissues for the presence of PrP^Sc^ is correlated with the scrapie disease state, but several exceptions have been reported in sheep with the ARQ/ARQ, VRQ/ARR, and VRQ/ARQ *PRNP* genotypes. [13], [34], [35] Though not addressed in the present study, it is possible that peripheral tissues from the ARQ/ARR sheep in the present study contain an accumulation of PrP^Sc^ that is below detection level for IHC and WB analysis but sufficient to transmit scrapie via IC sub-passage to a highly susceptible host recipient like a transgenic mouse overexpressing ovine PrP.

Sheep with genotypes conferring resistance to scrapie often have an incomplete distribution of PrP^Sc^ or do not have detectable PrP^Sc^ in lymphoid tissues. Rare natural cases of classical scrapie in ARR/ARR sheep have been reported. In these cases, PrP^Sc^ was detected in the CNS, but not in lymph node or tonsil tissue, [36] similar to the results obtained with IC inoculated ARQ/ARR sheep in the present study. Furthermore, in IC inoculated ARR/ARR sheep, attack rates are low, incubation periods are long, and PrP^Sc^ accumulation is not detected in the lymphoid system. [37] Similar findings have been reported for a single AHQ/ARR sheep IC inoculated with CH1641 scrapie and three sheep IC inoculated with BSE where PrP^Sc^ accumulated in brain, but not spleen. [38] At least in some instances, experimental oral inoculation of ARQ/ARH sheep also may accumulate PrP^Sc^ in the brain without detectable PrP^Sc^ in lymphoid tissues. [20] Prion disease induced in hamsters by intralingual (IL) inoculation resulted in progressive PrP^Sc^ accumulation in the brainstem following initial detection in the hypoglossal nucleus. [39] As in cases of scrapie in ARR/ARR sheep, PrP^Sc^ could not be detected in the LRS of these hamsters. Since both the CNS and the LRS have readily detectable PrP^Sc^ in scrapie susceptible sheep (ARQ/ARQ) following IL inoculation, [21] it appears that genetic resistance, and not route of exposure, plays the pivotal role in determining PrP^Sc^ propagation and accumulation in lymphoid organs.

Although ARQ/ARR sheep have enhanced resistance to scrapie, [12], [40] the present study documents the susceptibility of ARQ/ARR sheep to classical scrapie (inoculum No. 13-7) by IC inoculation, whereas oral inoculation of the same agent failed to transmit scrapie to neonatal ARQ/ARR sheep. [14] This is in contrast to work in the UK describing successful experimental oral inoculation of ARQ/ARR sheep that results in lymphoid involvement that is minimal, inconsistent, and late in the incubation period. [41] It is possible that the discordant results are due to the use of different scrapie isolates for the present study as compared to those previously reported. The drastically limited lymphoid involvement in the VRQ/ARR sheep inoculated in this study as compared to wide distribution in VRQ/ARR sheep orally inoculated in the UK could be due either to scrapie strain differences, other genetic factors outside of *PRNP*, or the unnatural route of inoculation (IC) used in the present study.
